# Acute Effects of Accelerated Eccentrics and Accentuated Eccentric Loading on Squat Performance and Lower-Limb Biomechanics

**DOI:** 10.3390/sports13120418

**Published:** 2025-12-01

**Authors:** Mingrui Zhang, Hao Zhou, Xiaoyan Xiang, Ran Wang

**Affiliations:** School of Athletic Performance, Shanghai University of Sport, Shanghai 200438, China; 2321811001@sus.edu.cn (M.Z.);

**Keywords:** accelerated eccentric loading, accelerated eccentrics, squat, strength, power, stretch-shortening cycle

## Abstract

This study aimed to compare the acute effects of three eccentric training strategies—constant resistance (CR), accentuated eccentric loading (AEL), and accelerated eccentrics (AE)—on the performance and biomechanical characteristics of the concentric phase of the squat, while maintaining a consistent squat depth. Twenty-four experienced resistance-trained male collegiate athletes (age: 21.92 ± 2.66 years; height: 175.88 ± 4.39 cm; body mass: 73.18 ± 8.08 kg) were recruited. A randomized crossover design was employed, where participants completed three squat protocols (eccentric load/concentric load/eccentric duration): AEL (90% 1RM/60% 1RM/2 s), CR (60% 1RM/60% 1RM/2 s), and AE (60% 1RM/60% 1RM/as fast as possible). Throughout the squats, kinematic and kinetic data were synchronously collected using an 8-camera 3D infrared motion capture system and two 3D force plates. The mean concentric barbell velocity in the AE condition was significantly higher than in both the AEL and CR conditions (*p* < 0.001). Furthermore, the AE condition demonstrated significant advantages in multiple biomechanical variables, including peak ground reaction force, as well as peak angular velocity and peak joint moments of the three lower limb joints (*p* < 0.05). With identical concentric loads and range of motion, increasing the velocity of the eccentric phase significantly enhances subsequent concentric performance and force output. In contrast, while the AEL strategy increases the mechanical load during the eccentric phase, its potentiating effect on concentric performance is relatively limited. These findings suggest that eccentric velocity may be a more critical variable than eccentric load in strength training.

## 1. Introduction

Resistance training (RT) is widely utilized to enhance muscle strength, power and athletic performance [1,2]. Traditional RT typically employs the same absolute load during both the concentric and eccentric phase of a movement. Skeletal muscle can generate substantially greater force during eccentric contractions than concentric contractions. A Bayesian meta-analysis estimated maximal eccentric strength is 40% higher than maximal concentric strength, typically ranging from 20% to 60% on average [3]. This implies that in constant resistance training, the training load is limited by the concentric capacity of the target muscles, potentially leading to a suboptimal stimulus during the eccentric phase [4].

Eccentric contractions produce greater tension at lower metabolic cost under the same load [5], giving eccentric training advantages for strength gains and musculo-tendinous remodeling. The high mechanical strain activates mechanosensitive pathways, increasing cross-sectional area and fascicle length and enhancing tendon stiffness and junction strength, thereby improving muscle–tendon mechanics [6,7]. Simultaneously, it enhances the recruitment capacity of high-threshold motor units, corticospinal excitability, and muscle group coordination, thereby optimizing force output and movement control [8].

Previous research has indicated that in multi-joint movements, optimizing the eccentric stimulus can enhance subsequent concentric power output through the Stretch-Shortening Cycle (SSC) mechanism, which promotes the release of elastic potential energy and provides neuromuscular advantages [9,10]. Rapid eccentric stretching increases neural drive during the subsequent concentric phase by elevating muscle pre-activation, enhancing muscle spindle-mediated stretch reflexes, and promoting the recruitment and discharge frequency of high-threshold motor units [10,11,12]. Consequently, overcoming the limitations imposed by concentric load on eccentric stimulus has become a key focus in recent sport science research.

Currently, accentuated eccentric loading (AEL) is a common strategy for optimizing eccentric stimulus. It involves applying a load during the eccentric phase that is greater than the concentric phase, often achieved using devices such as weight releasers, elastic bands, or dumbbells [13,14,15,16]. Multiple studies have confirmed that AEL training helps to increase concentric barbell velocity and power output [17,18,19,20], with potential underlying mechanisms including an enhanced muscle stretch reflex and the storage and release of energy in elastic components. However, a greater eccentric load often compels the trainee to reduce eccentric velocity to maintain control over the movement [21]. This may impair the efficiency of the SSC, thereby counteracting the potentiating effects on concentric performance. Concurrently, the higher mechanical stress and reliance on specialized equipment also limit its practical application.

In contrast, the accelerated eccentrics (AE) strategy enhances the eccentric stimulus by intentionally increasing eccentric velocity under conventional loads. Research has shown that a faster eccentric velocity can acutely or in the short-term increase concentric velocity and power [22,23,24]. The proposed mechanisms for this include higher levels of muscle activation, more efficient SSC utilization, and an enhanced stretch reflex [25,26,27]. Current evidence indicates that moderately prolonging the eccentric phase can confer advantages for muscle hypertrophy by increasing time under tension and muscle fibre strain; even when overall hypertrophic gains are similar, different contraction tempos may lead to differences in the regional distribution of muscle growth. Therefore, when the eccentric phase is excessively slow, it may not only reduce the efficiency of SSC utilisation but also shift training adaptations from primarily dynamic performance enhancement toward hypertrophy-dominant outcomes [28]. The AE strategy is less dependent on specialized equipment and involves a simpler procedure, making it not only easy to implement in conventional training settings but also featuring movement characteristics that more closely resemble the high-velocity eccentric scenarios found in actual sports. Although research has suggested that AE may produce acute potentiation effects similar to AEL [29], there are currently few studies that have directly compared the two. More importantly, previous research has often neglected to strictly control for squat depth. Variations in the range of motion can act as a confounding variable, obscuring the true effects of the eccentric strategy and limiting the practical application of eccentric training.

Therefore, the purpose of this study was to directly compare the acute effects of CR, AEL, and AE strategies on concentric squat performance and biomechanical characteristics under strictly controlled squat depth conditions. We hypothesized that, compared to CR and AEL, the AE protocol would elicit a higher concentric barbell velocity and greater lower-limb joint moment output. This research aims to provide a theoretical basis and practical guidance for athletes and coaches to develop more solid and effective resistance training programs.

## 2. Materials and Methods

### 2.1. Participants

An a priori power analysis (G*Power 3.1.9.6; effect size d = 0.25; α = 0.05; power = 0.80) indicated a required sample size of 15 participants. To account for potential invalid trials and individual variability, a total of 24 experienced resistance-trained male collegiate athletes were recruited for this study. The inclusion criteria were as follows: (1) male, aged 18–30 years; (2) back-squat 1RM exceeding 1.5 times their body mass; and (3) at least two years of resistance-training experience with a minimum of three regular training sessions per week. The exclusion criteria were (1) any history of lower-limb injuries or pain that could affect resistance training within the last three months; (2) cardiopulmonary disease; and (3) any history of lower-limb surgery. All participants were informed of the study’s purpose and procedures and signed an informed consent form prior to participation. The baseline characteristics of the participants are presented in Table 1.

### 2.2. Study Design

This study utilized a within-subjects, randomized crossover design. Participants first completed a standardized warm-up protocol, which included 5 min of cycling, foam rolling, dynamic stretching (world’s greatest stretch, knee-hug with calf raise, lateral leg cradle), and light-weight barbell squats. Subsequently, they performed three squat conditions (CR, AEL, and AE) in a randomized and counterbalanced order to compare the acute effects of different eccentric strategies on concentric performance and biomechanical variables. Randomization was performed using computer-generated sequences to ensure that each condition occurred equally often in each testing position. Each condition consisted of three repetitions [30], with a 30-s rest between repetitions and a 3–5 min rest between conditions. To minimize the interference from residual fatigue and learning effects, the formal testing session was conducted at least 48 h after the familiarization and 1RM testing sessions. The entire training and testing process was supervised by a certified strength and conditioning specialist (NSCA-CSCS) to ensure proper technique and participant safety.

### 2.3. Testing Procedures

The concentric load was identical across all three conditions, set at 60% of 1RM, and participants were instructed to perform the concentric phase of the squat with maximal effort and as fast as possible in every trial. To reinforce intent, we provided velocity feedback after each repetition and delivered standardized verbal encouragement throughout the sets. In the CR condition, the eccentric load was the same as the concentric load (60% 1RM), and the eccentric duration was controlled at 2 s using a metronome [31,32]. In the AEL condition, weight releasers were attached to both ends of the barbell, increasing the total eccentric load to 90% 1RM (60% 1RM from the barbell + 30% 1RM from the weight releasers), with the eccentric duration also controlled at 2 s. In the AE condition, the eccentric load was the same as the concentric load (60% 1RM), but participants were instructed to complete the eccentric phase as quickly as possible, no specific descent velocity was prescribed [33]. The range of motion was standardized using an individualized elastic band set as a tactile cue (knee flexion until the anterior thigh was parallel to the ground). A linear position transducer (GymAware, Canberra, Australia) was used throughout the session to provide real-time monitoring and immediate feedback on the eccentric/concentric velocity and duration of the squat. The average of the three squats in each group was used for analysis.

### 2.4. Data Collection and Processing

#### 2.4.1. Data Collection

**Figure 1:**
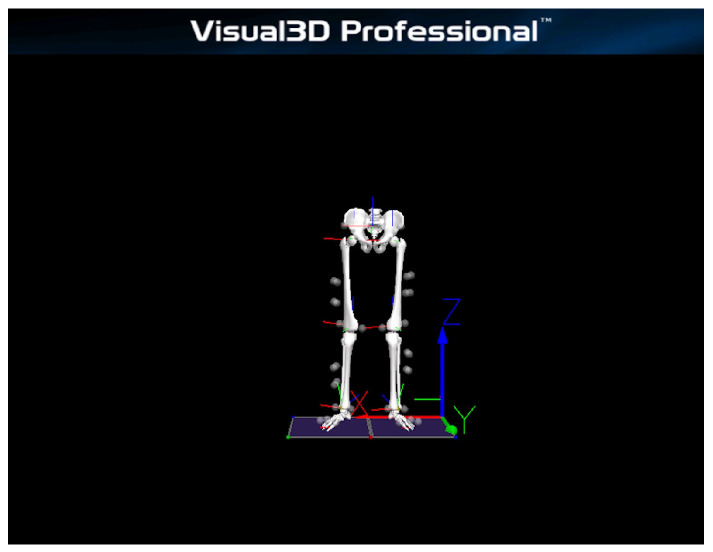
Schematic of reflective marker placement.

An eight-camera 3D infrared motion-capture system (Miqus, Qualisys, Göteborg, Sweden) was used to capture kinematic signals of the pelvis and lower limbs at a sampling frequency of 200 Hz. Following the CAST protocol, 40 reflective markers were placed on the following anatomical landmarks: anterior superior iliac spines (ASIS), posterior superior iliac spines (PSIS), lateral mid-thighs, medial and lateral femoral epicondyles, lateral mid-shanks, medial and lateral malleoli, calcanei, and the first, second, and fifth metatarsophalangeal joints. Two additional markers were placed on each end of the barbell to track its displacement (Figure 1). To ensure consistent placement, a single experienced operator placed all markers across sessions and conditions using a standardized protocol. Two 3D force plates (9260AA6, Kistler, Winterthur, Switzerland) were used record ground reaction forces at a sampling frequency of 1000 Hz. All kinematic and kinetic data were synchronized and collected using Qualisys Track Manager (QTM) 2024.1 software.

#### 2.4.2. Data Processing

**Figure 2:**
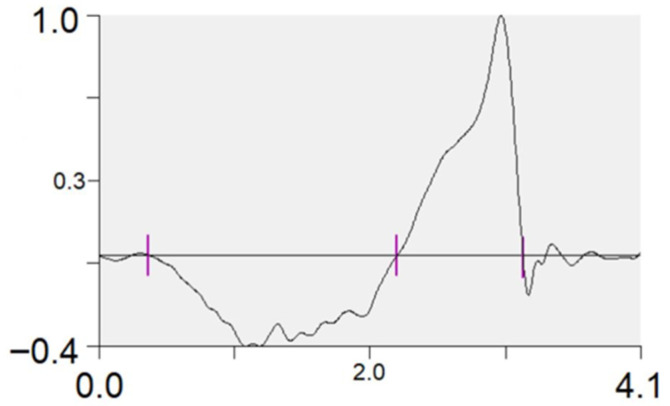
Phase delineation based on the vertical velocity of the center of mass.

Reflective markers were identified during static and dynamic trials using Qualisys Track Manager 2024.1, and the trajectories were exported as C3D files. A biomechanical model of the pelvis and lower limbs was created in Visual3D (v6.01.36, HAS-Motion, Kingston, ON, Canada). A fourth-order recursive Butterworth low-pass filter with a cutoff frequency of 15 Hz was applied to the raw kinematic and ground reaction force data, The cutoff frequency was determined using residual analysis during pilot testing [34,35]. Lower limb joint biomechanical variables and performance metrics were then calculated from the filtered signals. Joint rotations were expressed using an X–Y–Z Cardan sequence, following the right-hand rule about the axes of each segment’s local coordinate system. All kinetic variables were normalized to body mass. Barbell velocity was calculated from the trajectories of reflective markers affixed to the barbell. The eccentric and concentric phases were delineated using the vertical velocity of the center of mass (COM): the end of the eccentric phase/start of the concentric phase was defined as the point where velocity was zero and transitioning from negative to positive, and the end of the concentric phase was when the velocity approached and stabilized at zero [36,37] (Figure 2). Because low-pass filtering can slightly shift zero-crossings, all phase boundaries were verified visually on time-aligned velocity, displacement, and vertical GRF traces; when ambiguous, the COM displacement nadir and the onset of positive net vertical impulse were used as secondary criteria.

### 2.5. Statistical Analysis

All statistical analyses were performed using IBM SPSS Statistics 26.0 (IBM Corp., Armonk, NY, USA), with the significance level set at *p* < 0.05. All data are presented as mean ± standard deviation (M ± SD). First, the Shapiro–Wilk test and Levene’s test were used to assess normality and homogeneity of variances, respectively. Thereafter, a one-way repeated-measures ANOVA was conducted to examine differences in performance variables (movement time, barbell displacement, and barbell velocity) and biomechanical characteristics (hip, knee, and ankle joint angular velocities and moments) among CR, AEL, and AE squat conditions. When a significant main effect was detected, Bonferroni post hoc tests were used for pairwise comparisons. If the assumption of sphericity was violated (Mauchly’s test, *p* < 0.05), the Greenhouse–Geisser correction was applied. Effect sizes were expressed as Cohen’s d (trivial < 0.20; 0.20 ≤ small < 0.50; 0.50 ≤ medium < 1.00; large ≥ 1.00) [38]. In addition to *p* values and effect sizes, 95% confidence intervals (95% CI) were calculated for condition means and for pairwise mean differences (based on Bonferroni-adjusted comparisons) to improve the interpretability and precision of the estimates.

## 3. Results

### 3.1. Performance

#### 3.1.1. Movement Time

Repeated-measures ANOVA indicated significant differences in movement time for both the eccentric phase (F = 268.976, *p* < 0.001) and the concentric phase (F = 28.171, *p* < 0.001). Post hoc comparisons showed that AE had a significantly shorter eccentric-phase duration than AEL (95% CI [−1.14, −0.82], *p* < 0.001, |d| = 3.236) and CR (95% CI [−1.13, −0.87], *p* < 0.001, |d| = 4.002). AE also had a significantly shorter concentric-phase duration than AEL (95% CI [−0.14, −0.07], *p* < 0.001, |d| = 1.442) and CR (95% CI [−0.08, −0.03], *p* < 0.001, |d| = 1.143). In addition, the concentric-phase duration was significantly longer in AEL than in CR (95% CI [0.01, 0.09], *p* = 0.021, |d| = 0.600).

#### 3.1.2. Barbell Displacement

Repeated-measures ANOVA indicated a significant difference among conditions in concentric-phase barbell displacement (F = 4.337, *p* = 0.019). Post hoc tests showed that the AEL condition exhibited a significantly greater concentric-phase displacement than the CR condition (95% CI [0.01, 0.03], *p* = 0.022, |d| = 0.572).

#### 3.1.3. Barbell Velocity

**Figure 3:**
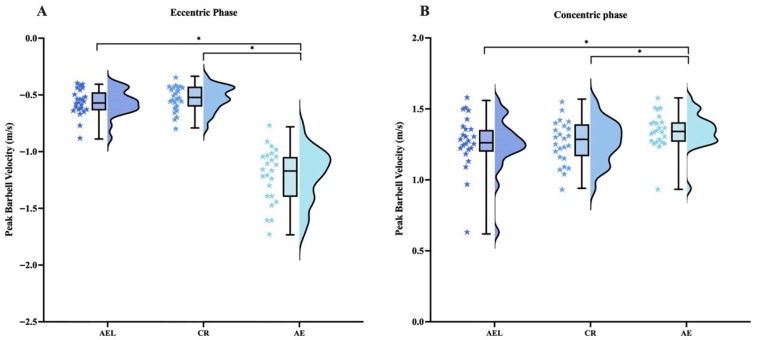
Peak barbell velocity during the eccentric (**A**) and concentric (**B**) phases under different loading conditions—accentuated eccentric loading (AEL), constant resistance (CR), accelerated eccentrics (AE). The colored stars represent the individual values of each participant. Error bars represent ±SD. * *p* < 0.05.

Repeated-measures ANOVA indicated significant differences among conditions in peak eccentric velocity (F = 185.512, *p* < 0.001), peak concentric velocity (F = 9.058, *p* < 0.001), mean eccentric velocity (F = 215.187, *p* < 0.001), and mean concentric velocity (F = 34.034, *p* < 0.001). Post hoc tests showed that the AE condition exhibited significantly higher peak eccentric velocity than AEL (95% CI [0.53, 0.76], *p* < 0.001, |d| = 2.978) and CR (95% CI [0.56, 0.80], *p* < 0.001, |d| = 2.896), higher peak concentric velocity than AEL (95% CI [0.03, 0.16], *p* = 0.006, |d| = 0.703) and CR (95% CI [0.02, 0.14], *p* = 0.004, |d| = 0.742), higher mean eccentric velocity than AEL (95% CI [0.30, 0.43], *p* < 0.001, |d| = 2.988) and CR (95% CI [0.31, 0.43], *p* < 0.001, |d| = 3.138), and higher mean concentric velocity than AEL (95% CI [0.06, 0.13], *p* = 0.001, |d| = 1.582) and CR (95% CI [0.04, 0.10], *p* = 0.001, |d| = 1.225) (Figure 3).

### 3.2. Kinematics

#### 3.2.1. Joint Angular Velocity

Repeated-measures ANOVA indicated significant differences among conditions in eccentric-phase peak angular velocities of the hip (F = 115.323, *p* < 0.001), knee (F = 151.706, *p* < 0.001), and ankle (F = 127.779, *p* < 0.001); eccentric-phase mean angular velocities of the hip (F = 228.159, *p* < 0.001), knee (F = 257.493, *p* < 0.001), and ankle (F = 189.528, *p* < 0.001); concentric-phase peak angular velocities of the Hip (F = 5.694, *p* = 0.007), knee (F = 10.057, *p* < 0.001) and ankle (F = 7.738, *p* = 0.003); and concentric-phase mean angular velocities of the hip (F = 14.054, *p* < 0.001), knee (F = 22.682, *p* < 0.001), and ankle (F = 14.523, *p* < 0.001).

**Figure 4:**
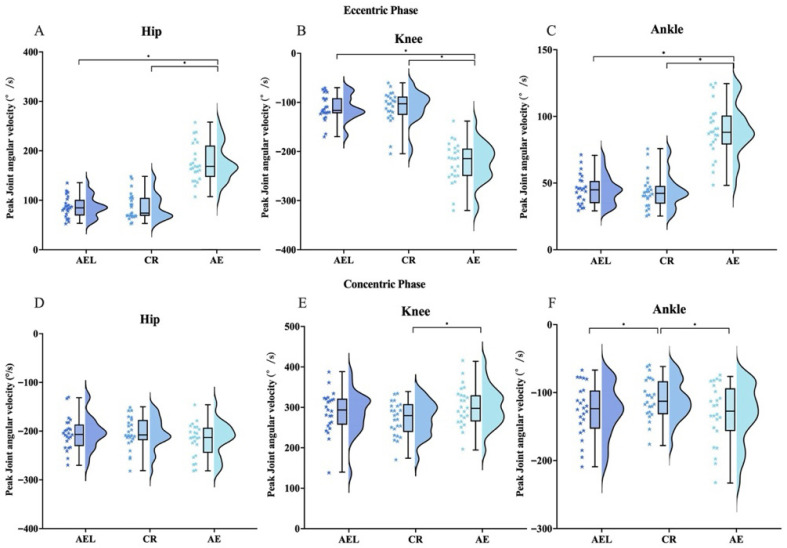
Peak Joint angular velocity across AEL, CR, and AE during the squat. Eccentric phase: panels (**A**–**C**); Concentric phase: panels (**D**–**F**). (**A**,**D**): Hip, (**B**,**E**): Knee, (**C**,**F**): Ankle. The colored stars represent the individual values of each participant. Error bars represent ±SD. * *p* < 0.05.

**Figure 5:**
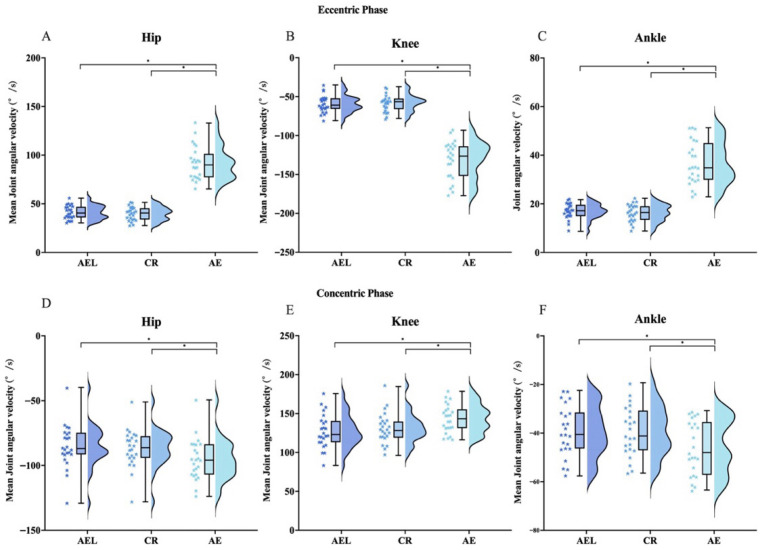
Mean Joint angular velocity across AEL, CR, and AE during the squat. Eccentric phase: panels (**A**–**C**); Concentric phase: panels (**D**–**F**). (**A**,**D**): Hip, (**B**,**E**): Knee, (**C**,**F**): Ankle. The colored stars represent the individual values of each participant. Error bars represent ±SD. * *p* < 0.05.

Post hoc tests showed that, during the eccentric phase, the AE condition exhibited significantly higher peak angular velocities than AEL and CR at the hip (95% CI [72.05, 106.65], *p* < 0.001, |d| = 2.724; 95% CI [67.26, 111.45], *p* < 0.001, |d| = 2.131), knee (95% CI [91.91, 132.70], *p* < 0.001, |d| = 2.902; 95% CI [90.61, 138.81], *p* < 0.001, |d| = 2.509), and ankle (95% CI [35.06, 53.00], *p* < 0.001, |d| = 2.587; 95% CI [35.78, 55.38], *p* < 0.001, |d| = 2.452), and significantly higher mean angular velocities than AEL and CR at the hip (95% CI [42.18, 59.67], *p* < 0.001, |d| = 3.069; 95% CI [43.28, 60.54], *p* < 0.001, |d| = 3.171), knee (95% CI [60.10, 82.89], *p* < 0.001, |d| = 3.306; 95% CI [61.34, 84.56], *p* < 0.001, |d| = 3.311), and ankle (95% CI [16.12, 23.38], *p* < 0.001, |d| = 2.869; 95% CI [16.67, 24.12], *p* < 0.001, |d| = 2.882). During the concentric phase, peak hip velocity in AE was significantly lower than in AEL (95% CI [1.87, 31.28], *p* = 0.24, |d| = 0.509) and CR (95% CI [16.39, 43.37], *p* = 0.30, |d| = 0.485); peak knee velocity in AE was significantly greater than in CR (95% CI [16.39, 43.37], *p* < 0.001, |d| = 1.077); peak ankle velocity in CR was significantly lower than in AEL (95% CI [−29.80, −2.552], *p* = 0.016, |d| = 0.626) and AE (95% CI [−31.80, −9.48], *p* < 0.001, |d| = 0.975). The AE condition also showed significantly higher mean angular velocities than AEL and CR at the hip (95% CI [5.23, 15.26], *p* < 0.001, |d| = 1.077; 95% CI [3.26, 14.49], *p* = 0.001, |d| = 0.833), knee (95% CI [9.61, 24.33], *p* < 0.001, |d| = 1.215; 95% CI [5.34, 20.02], *p* = 0.001, |d| = 0.910), and ankle (95% CI [2.73, 9.58], *p* < 0.001, |d| = 0.946; 95% CI [2.14, 10.05], *p* = 0.002, |d| = 0.812) (Figure 4 and Figure 5).

#### 3.2.2. Joint Angular Range of Motion

Repeated-measures ANOVA indicated that no significant differences were found among conditions in eccentric-phase peak angular velocities of the hip (F = 4.412, *p* = 0.059), knee (F = 3.501, *p* = 0.050), and ankle (F = 0.400, *p* = 0.673). Similarly, no significant differences were observed in concentric-phase peak angular velocities of the hip (F = 3.469, *p* = 0.052), knee (F = 0.277, *p* = 0.681), and ankle (F = 1.547, *p* = 0.224).

### 3.3. Kinetics

#### 3.3.1. Ground Reaction Forces (GRF)

**Figure 6:**
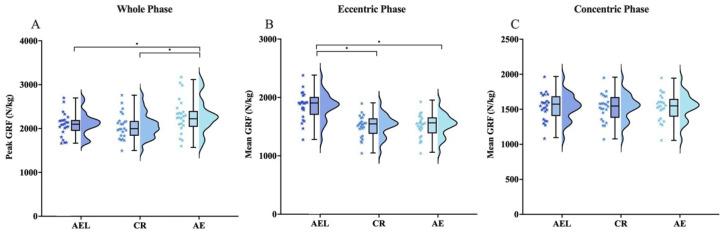
GRF across AEL, CR, and AE squat conditions. All values are normalized to body mass (N/kg). (**A**) Peak GRF across the whole phase; (**B**) mean eccentric GRF; (**C**) mean concentric GRF. The colored stars represent the individual values of each participant. Error bars represent ±SD. * *p* < 0.05.

Repeated-measures ANOVA indicated significant differences among conditions in overall peak force (F = 23.634, *p* < 0.001) and mean eccentric force (F = 1886.742, *p* < 0.001). Post hoc tests showed that the AE condition exhibited a significantly greater overall peak force than AEL (95% CI [0.97, 3.51], *p* < 0.001, |d| = 0.741) and CR (95% CI [1.57, 3.91], *p* < 0.001, |d| = 0.835). The AEL condition showed a significantly greater mean eccentric force than CR (95% CI [4.14, 4.62], *p* < 0.001, |d| = 3.631) and AE (95% CI [4.09, 4.62], *p* < 0.001, |d| = 3.593) (Figure 6).

#### 3.3.2. Joint Moments

**Figure 7:**
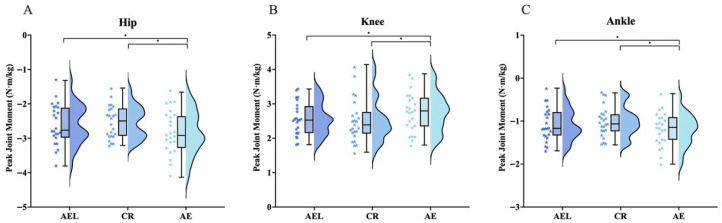
Peak joint moments across the whole phase during AEL, CR, and AE squats. Values are normalized to body mass (N·m/kg). (**A**) Hip, (**B**) Knee, (**C**) Ankle. The colored stars represent the individual values of each participant. Error bars represent ±SD. * *p* < 0.05.

Repeated-measures ANOVA indicated significant differences among conditions in peak joint moments and in mean eccentric joint moments for the hip (F = 7.110, *p* = 0.002; F = 24.708, *p* < 0.001), knee (F = 7.588, *p* = 0.001; F = 29.303, *p* < 0.001), and ankle (F = 5.839, *p* = 0.005; F = 14.669, *p* < 0.001). Post hoc tests showed that the AE condition exhibited significantly greater peak joint moments than AEL and CR at the hip (95% CI [0.02, 0.47], *p* = 0.033, |d| = 0.409; 95% CI [0.10, 0.47], *p* = 0.002, |d| = 0.505), knee (95% CI [0.17, 0.34], *p* = 0.027, |d| = 0.338; 95% CI [0.05, 0.41], *p* = 0.009, |d| = 0.391), and ankle (95% CI [0.01, 0.24], *p* = 0.040, |d| = 0.328; 95% CI [0.03, 0.34], *p* = 0.017, |d| = 0.509). The AEL condition showed significantly greater mean eccentric joint moments than CR and AE at the hip (95% CI [0.17, 0.43], *p* < 0.001, |d| = 0.726; 95% CI [0.11, 0.31], *p* < 0.001, |d| = 0.493), knee (95% CI [0.16, 0.31], *p* < 0.001, |d| = 0.709; 95% CI [0.12, 0.27], *p* < 0.001, |d| = 0.639), and ankle (95% CI [0.11, 0.27], *p* < 0.001, |d| = 0.704; 95% CI [0.02, 0.24], *p* = 0.013, |d| = 0.494) (Figure 7). See Table 2 for details.

## 4. Discussion

The principal finding of this study is that AE enhances concentric-phase performance and kinetic outputs more effectively than both CR and AEL. Specifically, AE produced a higher mean concentric barbell velocity, greater lower-limb joint angular velocities, and larger peak joint moments. In contrast, although AEL markedly increased the external load during the eccentric phase, its acute performance-enhancing effect on the subsequent concentric phase was limited.

A key strength of this study is the rigorous control of squat depth. By ensuring that eccentric-phase barbell displacement and joint range of motion (ROM) were closely matched across the three conditions, we effectively eliminated movement amplitude as a potential confounding variable. Prior studies have often failed to control or report squat depth [27,28], and evidence indicates that faster eccentric velocities may coincide with a greater squat depth [28], which can, in itself, elevate peak velocity by lengthening the acceleration distance and may even alter lower-limb force-production patterns. Accordingly, the present findings more convincingly attribute the between-condition differences in concentric performance to variations in eccentric velocity rather than to differences in squat depth, thereby strengthening the internal validity of the results.

Our findings clearly demonstrate that the AE protocol, by shortening the eccentric duration, significantly increased barbell and joint angular velocities during the eccentric phase, which translated into superior concentric performance. This finding supports the theory that faster eccentric contractions facilitate subsequent concentric bursts. The underlying physiological mechanism is primarily attributed to the more efficient utilization of the SSC. A high-speed eccentric movement enables musculotendinous units to store and rapidly release greater elastic potential energy [29], while also allowing muscle fibers to operate within a more favorable range of the force–velocity curve, thereby enhancing force output at the transition point from eccentric to concentric. Moreover, rapid muscle stretching more strongly activates the stretch reflex, which can shorten the amortization phase and improve neuromuscular efficiency [30,31]. Our kinetic data corroborate this interpretation: the AE condition produced a higher peak GRF and greater peak moments at all three lower-limb joints, directly reflecting superior joint moment production. Furthermore, high-speed eccentric loading is known to preferentially recruit fast-twitch muscle fibers and enhance motor unit excitability, both of which are crucial for explosive performance. Conversely, slower movement tempos can reduce concentric force and power while diminishing SSC benefits by decreasing type IIx fiber recruitment. It is worth contrasting these results with the findings of Chan et al. [32], who compared unloaded squats at different eccentric velocities. They found that rapid eccentrics significantly increased concentric hip and knee extension moments but had minimal influence on plantar flexion moments. This suggests that under low external loads, the ankle joint may primarily serve a postural stabilizing function. Our study indicates, however, that when a significant external load is applied, the SSC benefits of a rapid eccentric contraction extend from proximal to distal segments, with the increase in ankle moment even surpassing that observed at the hip and knee.

In this study, the AEL condition did not demonstrate the anticipated enhancement of concentric performance. While mean joint moments and GRFs in the eccentric phase increased significantly with the heavier load, these increases did not translate into a faster concentric velocity or greater peak concentric joint moments. A plausible explanation is that when faced with a higher eccentric load (90% 1RM), participants adopted an active “braking” strategy to maintain movement stability and control. This strategy not only constrained descent velocity but also dampened rapid musculotendinous lengthening and, consequently, limited exploitation of the SSC, precluding sufficient elastic energy storage and stretch-reflex potentiation.

Previous work has shown that, as eccentric speed increases, both eccentric peak force and RFD change accordingly. The altered patterns of joint moment and power contribution suggest that higher levels of movement control and technical stability are required [39]. It is therefore conceivable that our participants reduced descent velocity in the AEL condition to preserve technique and balance under the heavier eccentric load. This interpretation is exemplified by the findings of Armstrong et al. [40], who reported that when AEL back squats were performed with a very slow eccentric phase, the resulting whole-body peak force was only 2% greater than that achieved during maximal-effort traditional squats, suggesting that markedly prolonging the eccentric duration may largely negate the potential mechanical advantage of AEL. The AEL effect depends not only on load magnitude but also on the ability to maintain sufficiently high eccentric speed while increasing load. This suggests that the benefits of AEL are not dictated by load magnitude alone, but rather hinge on whether the increased load is paired with a sufficiently high eccentric velocity. The favorable effects of AEL reported in previous studies may have been driven in part by uncontrolled, load-induced increases in squat depth and knee flexion, factors known to redistribute joint moments across the lower limb [41]. This further underscores the need for future AEL studies to explicitly report and strictly control squat depth. Notably, although AEL exerted minimal influence on overall concentric barbell velocity, we observed modest increases in peak joint angular velocities at the knee and ankle. This pattern may reflect the relatively high strength levels of our participants, as stronger individuals may require a greater magnitude of eccentric overload to reach the loading threshold that elicits optimal concentric performance [42].

### Limitations

1. Although an elastic band was used as an external reference to standardize squat depth and ROM across conditions, this approach may have introduced small between-subject differences in the actual movement amplitude, which could have slightly affected the kinematic and kinetic outputs.
2. Upper-limb contributions were not explicitly modeled. Although the squat task and grip were standardized so that the arms served only to stabilize the barbell, a small influence of upper-limb involvement on the lower-limb outcomes cannot be completely ruled out.

## 5. Conclusions

Under conditions of matched concentric load and movement amplitude, the AE strategy improved concentric-phase barbell velocity and overall force production more effectively than both CR and AEL. Although AEL increased the mechanical load during the eccentric phase, its potentiating effect on concentric performance was limited. Therefore, for athletes and practitioners aiming to enhance explosive performance, emphasizing a rapid eccentric phase may be a safer and more efficient training strategy than simply increasing the eccentric load. Nevertheless, this tentative recommendation is derived from acute responses in resistance-trained individuals and does not directly address long-term adaptations or injury risk, so long-term, well-controlled intervention studies in athletes with different training levels and risk profiles are still required.

## Figures and Tables

**Table 1:** Participant characteristics.

Age (yrs)	Height (cm)	Body Mass (kg)	Body Fat (%)	Back-Squat 1RM (kg)
21.92 ± 2.66	175.88 ± 4.39	73.18 ± 8.08	14.66 ± 3.61	134.29 ± 18.78

**Table 2:** Biomechanical variables (mean ± SD) during AEL, CR, and AE squat conditions.

Variable	Phase	AEL	CR	AE	*p*-Value
Movement time (s)	Eccentric	1.84 ± 0.29 ^c^	1.86 ± 0.24 ^c^	0.85 ± 0.12	<0.001 *
	Concentric	0.85 ± 0.13 ^cb^	0.80 ± 0.09 ^c^	0.74 ± 0.08	<0.001 *
Barbell displacement (m)	Eccentric	0.55 ± 0.06	0.55 ± 0.07	0.56 ± 0.07	0.138
	Concentric	0.59 ± 0.06 ^b^	0.58 ± 0.06	0.59 ± 0.06	0.019 *
Peak barbell velocity (m/s)	Eccentric	0.57 ± 0.11 ^c^	0.53 ± 0.11 ^c^	1.21 ± 0.24	<0.001 *
	Concentric	1.26 ± 0.19 ^c^	1.27 ± 0.15 ^c^	1.35 ± 0.13	<0.001 *
Mean barbell velocity (m/s)	Eccentric	0.33 ± 0.05 ^c^	0.33 ± 0.05 ^c^	0.70 ± 0.14	<0.001 *
	Concentric	0.68 ± 0.09 ^c^	0.70 ± 0.09 ^c^	0.77 ± 0.09	<0.001 *
Hip ROM (°)	Eccentric	80.58 ± 7.27	79.15 ± 9.39	82.39 ± 9.25	0.059
	Concentric	80.47 ± 7.99	77.55 ± 7.95	79.52 ± 8.69	0.052
Knee ROM (°)	Eccentric	88.50 ± 20.12	87.97 ± 21.52	90.92 ± 20.96	0.050
	Concentric	91.69 ± 20.23	91.19 ± 21.68	92.32 ± 21.99	0.681
Ankle ROM (°)	Eccentric	27.49 ± 5.60	26.96 ± 6.11	27.21 ± 5.29	0.673
	Concentric	31.26 ± 7.27	29.48 ± 7.00	31.01 ± 7.26	0.224
Peak hip angular velocity (°/s)	Eccentric	86.69 ± 21.28 ^c^	86.68 ± 26.97 ^c^	176.03 ± 38.39	<0.001 *
	Concentric	203.91 ± 33.10	204.40 ± 32.65	216.23 ± 34.37	<0.001 *
Peak knee angular velocity (°/s)	Eccentric	111.76 ± 26.30 ^c^	109.35 ± 33.75 ^c^	224.06 ± 41.88	<0.001 *
	Concentric	286.48 ± 51.13	274.07 ± 42.40 ^c^	301.11 ± 49.33	0.056
Peak ankle angular velocity (°/s)	Eccentric	44.94 ± 11.06 ^c^	43.39 ± 12.61 ^c^	88.97 ± 19.67	<0.001 *
	Concentric	126.66 ± 38.76 ^b^	110.48 ± 29.69 ^c^	131.12 ± 43.52	0.003 *
Mean hip angular velocity (°/s)	Eccentric	40.98 ± 6.91 ^c^	40.00 ± 6.91 ^c^	91.91 ± 17.25	<0.001 *
	Concentric	85.42 ± 16.65 ^c^	86.79 ± 14.63 ^c^	95.67 ± 15.86	0.001 *
Mean knee angular velocity (°/s)	Eccentric	59.52 ± 10.96 ^c^	58.07 ± 10.54 ^c^	131.02 ± 24.06	<0.001 *
	Concentric	127.12 ± 21.79 ^c^	131.40 ± 19.62 ^c^	144.08 ± 18.24	<0.001 *
Mean ankle angular velocity (°/s)	Eccentric	16.90 ± 3.17 ^c^	16.26 ± 3.55 ^c^	36.65 ± 8.82	<0.001 *
	Concentric	38.93 ± 10.63 ^c^	38.99 ± 10.22 ^c^	45.08 ± 11.17	<0.001 *
Peak GRF (N/kg)	Whole movement	28.75 ± 2.04 ^c^	28.25 ± 2.72 ^c^	30.99 ± 3.76	<0.001 *
Mean GRF (N/kg)	Eccentric	25.18 ± 1.37 ^bc^	20.81 ± 1.01	20.84 ± 1.02	<0.001 *
	Concentric	20.91 ± 0.90	20.93 ± 0.99	20.99 ± 1.03	0.349
Peak hip joint moment (Nm/kg)	Whole movement	2.56 ± 0.55 ^c^	2.52 ± 0.47 ^c^	2.80 ± 0.63	0.002 *
Peak knee joint moment (Nm/kg)	Whole movement	2.57 ± 0.47 ^c^	2.51 ± 0.63 ^c^	2.75 ± 0.56	0.001 *
Peak ankle joint moment (Nm/kg)	Whole movement	1.09 ± 0.37 ^c^	1.03 ± 0.34 ^c^	1.21 ± 0.38	0.005 *
Mean hip joint moment (Nm/kg)	Eccentric	1.59 ± 0.46 ^bc^	1.29 ± 0.35	1.38 ± 0.38	<0.001 *
	Concentric	1.65 ± 0.44	1.69 ± 0.40	1.68 ± 0.40	<0.001 *
Mean knee joint moment (Nm/kg)	Eccentric	1.76 ± 0.33 ^bc^	1.52 ± 0.33	1.56 ± 0.28	<0.001 *
	Concentric	1.54 ± 0.30	1.52 ± 0.30	1.50 ± 0.30	0.609
Mean ankle joint moment (Nm/kg)	Eccentric	0.63 ± 0.30 ^bc^	0.43 ± 0.24	0.49 ± 0.24	0.589
	Concentric	0.64 ± 0.31	0.65 ± 0.30	0.73 ± 0.27	0.100

Note: Values are mean ± SD. “b/c” indicates a significant difference from CR/AE. * *p* < 0.05.

## Data Availability

The data presented in this study are available on request from the corresponding author due to privacy restrictions.
